# Supplementary material for: In Silico Identification and Biological Evaluation of Antioxidant Food Components Endowed with Human Carbonic Anhydrase IX and XII Inhibition
Source: Antioxidants (Basel). 2020 Aug 21;9(9):775. doi: 10.3390/antiox9090775 (PMC7555330; doi:10.3390/antiox9090775)
Supplement: Supplementary file 1 [file antioxidants-09-00775-s001.pdf]

Supplementary Materials for

In silico identification and biological evaluation of antioxidant food components endowed with IX and XII *h*CA inhibition

Giosuè Costa [1,2#], Annalisa Maruca [1,2#], Roberta Rocca [2,3], Francesca Alessandra Ambrosio [1], Emanuela Berrino[4], Fabrizio Carta [4], Francesco Mesiti [1,2], Alessandro Salatino [3], Delia Lanzillotta [3], Francesco Trapasso [3], Anna Artese [1,2], Stefano Alcaro [1,2*] and Claudiu T. Supuran [4]

[1] Dipartimento di Scienze della Salute, Università "Magna Græcia" di Catanzaro, Campus "S. Venuta", Viale Europa, 88100, Catanzaro, Italy; gcosta@unicz.it (G.C.); maruca@unicz.it (A.M.); ambrosio@unicz.it (F.A.A); francesco.mesiti@studenti.unicz.it (F.M); artese@unicz.it (A.A.); alcaro@unicz.it (S.A)

[2] Net4Science Academic Spin-Off, Università "Magna Græcia" di Catanzaro, Campus "S. Venuta", Viale Europa, 88100, Catanzaro, Italy; rocca@unicz.it (R.R.)

[3] Dipartimento di Medicina Sperimentale e Clinica, Università "Magna Græcia" di Catanzaro, Campus "S. Venuta", Viale Europa, 88100, Catanzaro, Italy; salatino@unicz.it (A.S); delialanzillotta@unicz.it (D.L.); trapasso@unicz.it (F.T)

[4] Dipartimento NEUROFARBA, Sezione di Scienze Farmaceutiche, Università degli Studi di Firenze, Sesto Fiorentino, Florence, Italy; emanuela.berrino@unifi.it (E.B.); fabrizio.carta@unifi.it (F.C); claudiu.supuran@unifi.it (C.T.S.)

* Correspondence: alcaro@unicz.it; Tel.+39 0961 3694198 (S. A.)

#These authors contributed equally.

Table of content

Figure S1. 2D chemical structures of the already approved inhibitors of both *h*CA isoforms.
Table S1. 2D chemical structures, G-score and ΔE values for each already approved inhibitor of the *h*CA XII isoform.
Table S2. 2D chemical structures, G-score and ΔE values for each already approved inhibitor of the *h*CA IX isoform.
Table S3. Name, FooDB ID, G-score and ΔE values of the 9 best dual *hits* related to both *h*CA isoforms.
Figure S2. 2D chemical structures of the best 9 selected *hits*.

Figure S1. 2D chemical structures of the already approved inhibitors of both *h*CA isoforms.

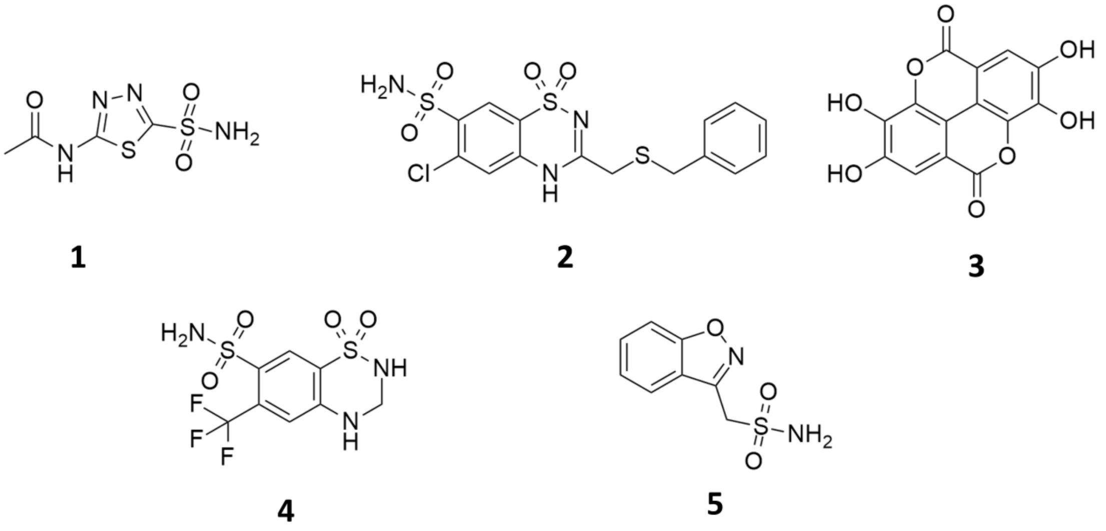

Table S1. 2D chemical structures, G-score and ΔE values for each already approved inhibitor of the *h*CA XII isoform.

**Compound**	**Name**	**G-Score** *(kcal/mol)*	**ΔE** *(kcal/mol)*
**1**	Acetazolamide	-4.56	-28.56
**2**	Benzthiazide	-5.62	-34,12
**3**	Ellagic acid	-5.51	-30.10
**4**	Hydroflumethiazide	-5.86	-28.30
**5**	Zonisamide	-5.34	-19.86

Table S2. 2D chemical structures, G-score and ΔE values for each already approved inhibitor of the *h*CA IX isoform.

**Compound**	**Name**	**G-Score** *(kcal/mol)*	**ΔE** *(kcal/mol)*
**2**	Benzthiazide	-4.96	-23.30
**3**	Ellagic acid	-5.18	-26.60
**4**	Hydroflumethiazide	-5.10	-12.57
**5**	Zonisamide	-5.22	-25.21

Table S3. Name, FooDB ID, G-score and ΔE values of the 9 best dual *hits* related to both *h*CA isoforms.

			***h*AC IX**		***h*AC XII**	
**Number**	**Name**	**FooDB ID**	**G-Score** *(kcal/mol)*	**ΔE** *(kcal/mol)*	**G-Score** *(kcal/mol)*	**ΔE** *(kcal/mol)*
**6**	(-)-Dehydrodiconiferyl Alcohol	FDB021188	-5.76	-37.89	-6.15	-43.75
**7**	13'-carboxy-α-tocopherol	FDB029121	-5.88	-35.86	-7.23	-58.88
**8**	8-Hydroxy-3-methoxy-1-methylanthraquinone-2-carboxylic acid	FDB016091	-7.10	-32.99	-8.42	-35.20
**9**	Albafuran A	FDB001381	-5.59	-35.25	-6.49	-33.59
**10**	Cartormin	FDB013856	-6.02	-45.64	-6.45	-62.46
**11**	Licoagroaurone	FDB014047	-5.50	-41.16	-6.18	-42.89
**12**	Lithospermic acid	FDB006174	-5.14	-23.42	-7.32	-31.82
**13**	Piperoic acid	FDB020410	-6.99	-40.69	-7.50	-51.16
**14**	Syringin	FDB011657	-5.12	-31.22	-6.12	-43.81

Figure S2. 2D chemical structures of the best 9 selected *hits*.

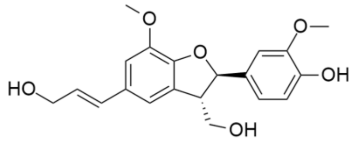

6

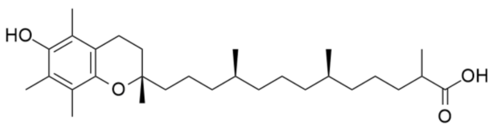

7

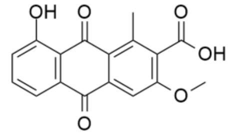

8

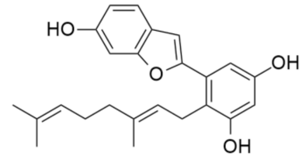

9

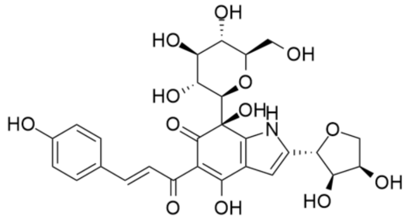

10

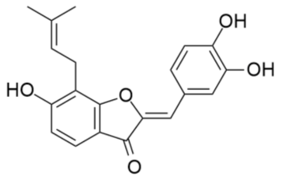

11

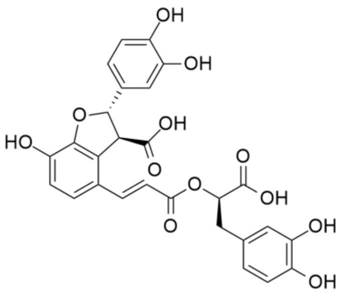

12

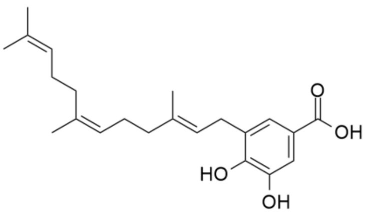

13

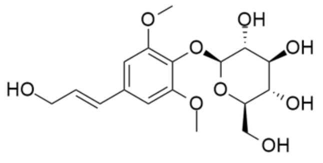

14
